# Cross Species Genomic Analysis Identifies a Mouse Model as Undifferentiated Pleomorphic Sarcoma/Malignant Fibrous Histiocytoma

**DOI:** 10.1371/journal.pone.0008075

**Published:** 2009-11-30

**Authors:** Jeffrey K. Mito, Richard F. Riedel, Leslie Dodd, Guy Lahat, Alexander J. Lazar, Rebecca D. Dodd, Lars Stangenberg, William C. Eward, Francis J. Hornicek, Sam S. Yoon, Brian E. Brigman, Tyler Jacks, Dina Lev, Sayan Mukherjee, David G. Kirsch

**Affiliations:** 1 Department of Pharmacology and Cancer Biology, Duke University Medical Center, Durham, North Carolina, United States of America; 2 Division of Medical Oncology, Department of Medicine, Duke University Medical Center, Durham, North Carolina, United States of America; 3 Department of Pathology, Duke University Medical Center, Durham, North Carolina, United States of America; 4 Department of Surgical Oncology, The University of Texas MD Anderson Cancer Center, Houston, Texas, United States of America; 5 Department of Pathology, The University of Texas MD Anderson Cancer Center, Houston, Texas, United States of America; 6 Department of Radiation Oncology, Duke University Medical Center, Durham, North Carolina, United States of America; 7 Department of Surgery, Massachusetts General Hospital and Harvard Medical School, Boston, Massachusetts, United States of America; 8 Division of Orthopaedic Surgery, Department of Surgery, Duke University Medical Center, Durham, North Carolina, United States of America; 9 Center for Cancer Research, Massachusetts Institute of Technology, Cambridge, Massachusetts, United States of America; 10 Howard Hughes Medical Institute, Chevy Chase, Maryland, United States of America; 11 Department of Statistical Sciences, Duke University, Durham, North Carolina, United States of America; 12 Institute for Genome Sciences and Policy, Duke University Medical Center, Durham, North Carolina, United States of America; Health Canada, Canada

## Abstract

Undifferentiated pleomorphic sarcoma/Malignant Fibrous Histiocytoma (MFH) is one of the most common subtypes of human soft tissue sarcoma. Using cross species genomic analysis, we define a geneset from the *LSL-Kras^G12D^; Trp53^Flox/Flox^* mouse model of soft tissue sarcoma that is highly enriched in human MFH. With this mouse geneset as a filter, we identify expression of the RAS target FOXM1 in human MFH. Expression of *Foxm1* is elevated in mouse sarcomas that metastasize to the lung and tissue microarray analysis of human MFH correlates overexpression of FOXM1 with metastasis. These results suggest that genomic alterations present in human MFH are conserved in the *LSL-Kras^G12D^; p53^Flox/Flox^* mouse model of soft tissue sarcoma and demonstrate the utility of this pre-clinical model.

## Introduction

Malignant Fibrous Histiocytoma was first described in the 1960s and quickly became the most commonly diagnosed adult soft tissue sarcoma [1]. Because these tumors do not appear to arise from histiocytes, the term Malignant Fibrous Histiocytoma has recently fallen out of favor and many pathologists now classify these tumors as undifferentiated pleomorphic sarcomas. Despite this change in nomenclature, undifferentiated pleomorphic sarcoma (referred to here as MFH) remains one of the most common adult soft tissue sarcomas encountered in the clinic. However, the cell(s) of origin of MFH is unknown. Indeed, some have suggested that MFH is a collection of undifferentiated mesenchymal tumors sharing a common morphology rather than a single clinical entity [2], [3], [4]. This debate could be clarified by identifying the cell(s) of origin of a mouse model for MFH. Whether MFH describes a cancer that is a single pathogenic entity or an undifferentiated state shared by several sarcoma subtypes, the survival of patients with MFH has not improved for decades. Therefore, identifying a mouse model of MFH may also lead to better treatments for patients with this diagnosis.

Human MFH is characterized by a propensity to metastasize to the lungs and by a range of histologic appearances including spindle and pleomorphic cells. Although these features are recapitulated in a mouse model of soft tissue sarcoma initiated by conditional mutations in *Kras* and *Trp53* [5], it is not clear whether this model is most similar to human MFH or another soft tissue sarcoma. Therefore, we sought to more accurately classify the sarcoma subtype for this mouse model using gene expression profiling.

## Methods

### Mouse Genotyping, Tumor Generation, and Determination of Metastatic Potential

Both mouse genotyping and generation of tumors was carried out as described previously [5] in accordance with Duke University and MIT Institutional Animal Care and Use Committee approved protocols. Sarcomas were induced in the lower left limb and allowed to grow until ∼200 mm^3^ in volume. Tumors were then surgically excised via amputation of the limb and animals followed for a minimum of 4 months to determine the metastatic potential of the primary tumor.

### RNA Isolation

RNA was extracted from *LSL-Kras^G12D^; Trp53^Flox/Flox^* tumors or normal muscle using TRIzol reagent (Invitrogen) and was purified using RNeasy mini kit (Qiagen).

### Microarray Processing and Analysis

Full details can be found in supplementary methods S1. Briefly, gene expression was determined using Affymetrix 430A 2.0 arrays (Affymetrix) as described in detail online (http://www.genome.duke.edu/cores/microarray/). CEL files were processed using the RMA algorithm [6], [7] to normalize the data. Genesets were identified using a signal-to-noise metric.

Human and Mouse datasets [8], [9], [10] were downloaded from GEO (GSE6461, GSE6481, GSE2553, and GDS1209), normalized with RMA (when appropriate). Genesets and array data were used in GSEA as described previously [11]. Classes were defined as one soft tissue sarcoma subtype versus controls (other sarcoma or normal muscle) present in their respective datasets.

### Database Accession Numbers

Microarray data was generated in conformity to MIAME guidelines and has been deposited in the GEO database under accession number GSE16779.

### Oncogenic Pathway Predictors

Human soft tissue sarcoma datasets [9], [10] were combined using ComBat [12] and normal tissue samples removed from the combined dataset. An oncogenic pathway classifier for Ras pathway activity was developed as described previously [13]. This classifier was used to compare undifferentiated pleiomorphic sarcoma/MFH samples (n = 29) against all other soft tissue sarcomas. Significance was determined using a non-parametric Mann-Whitney test.

### Histology and Immunohistochemistry and Image Analysis

All human samples were obtained from tissue repositories at Duke and MD Anderson. These samples were used in accordance with Duke and MD Anderson Cancer Center Institutional Review Board (IRB) approved protocols under a waiver of consent. Five micron thick sections were cut from formalin fixed paraffin embedded samples. Samples were subjected to standard hematoyxlin and eosin staining or immunohistochemistry. Immunohistochemistry was performed with the following antibodies: phospho-ERK (Invitrogen 29-2389) and FOXM1 (Abcam ab47808), using the Vectastain ABC Rabbit IgG kit with Vectastain Elite ABC Reagent (Vector Labs).

Brightfield images of slides taken at 40x were used for analysis using Image Pro AMS v6.1. The counting module was trained using both positive and negative nuclear staining for phospho-ERK. A minimum of 3000 nuclei were counted per sample and a ratio between total nuclei with positive nuclei to total nuclei was determined using a minimal and maximal area of 100 and 1000 pixels respectively.

### Tissue Microarrays (TMAs)

TMAs were generated at MD Anderson Cancer Center and contained a clinically annotated set of 214 soft tissue sarcoma samples including: 166 MFH/Unclassified sarcomas, 19 synovial sarcomas, 6 leiomyosarcomas, 8 pleomorphic liposarcomas, 8 myxoid liposarcomas, 6 atypical lipomatous tumors, and 1 dedifferentiated liposarcoma. TMAs were stained as above and scored semiquantitatively on a scale from 0-3+ by a musculoskeletal pathologist (L.D.) blinded to patient outcome. Scores were correlated with both diagnosis and clinical outcome.

### Statistical Analysis of TMAs

Scoring of TMAs was correlated with diagnosis, and metastasis-free survival.

Correlation of immunohistochemical staining with diagnosis was tested for normality using a chi-square test. This did not reach statistical significance, therefore comparison between MFH and other soft tissue sarcomas was performed using the non-parametric Mann-Whitney test.

Metastasis free survival analysis was performed on MFH samples comparing 3+ staining to 0-2+ staining. Survival was determined by Kaplan-Meier analysis.

## Results

### Cross-Species Genomic Analysis of *LSL-KRas^G12D^;Trp53^Flox/Flox^* Mouse Model of Soft Tissue Sarcoma

**Figure 1:**
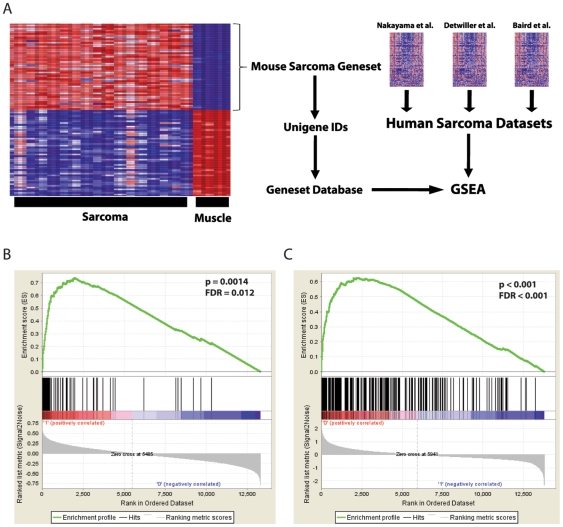
Cross-species genomic comparison. *A*, Schematic for cross species genomic comparison using GSEA. *B*, The *LSL-Kras^G12D^; Trp53^FloxFlox^* sarcoma geneset (Table S3) is highly enriched in human MFH in the Nakayama et al. soft tissue sarcoma dataset [10] [p = 0.0014; False Discovery Rate (FDR) = 0.012; Enrichment Score (ES) = 0.74; Normalized Enrichment Score (NES) = 2.09]. *C*, Conversely, a human MFH geneset (Table S4) is strongly enriched in the mouse model of soft tissue sarcoma (p<0.001, FDR<0.001, ES = 0.637 NES = 2.78).

We hypothesized that genes most differentially expressed between mouse sarcoma and normal muscle would provide a useful molecular signature to interrogate human sarcoma datasets (Fig. 1A). To test this approach, we first analyzed published gene expression data from a previously validated mouse model of synovial sarcoma [14]. After identifying a geneset of 100 genes highly overexpressed in synovial sarcoma compared to normal muscle (Table S1) we used Gene Set Enrichment Analysis (GSEA) [11] to probe gene expression data from three studies of human soft tissue sarcomas [8], [9], [10]. We demonstrated strong statistical enrichment (p<0.001, FDR<0.02) of this geneset in human synovial sarcomas, but not in other subtypes of soft tissue sarcoma (Fig. S1, Table S2). This result is in agreement with GSEA for the mouse model of synovial sarcoma that was previously reported [14].

Having validated this approach, we identified a geneset of 100 genes highly overexpressed in the *LSL-Kras^G12D^; Trp53^Flox/Flox^* mouse model of soft tissue sarcoma (n = 17) compared to normal muscle (n = 4) (Table S3). When this geneset was analyzed in the three human datasets of soft tissue sarcoma [8], [9], [10], only MFH samples showed statistical enrichment (p = 0.001; FDR = 0.012) (Table 1, Fig. 1B). Enrichment was seen in all three datasets [8], [9], [10], which represent 325 sarcoma samples and two types of array platforms. Moreover, genesets derived from human MFH (Table S4) also enriched in the mouse sarcoma data (p<0.001; FDR<0.001) (Fig. 1C). These data indicate that this mouse sarcoma model and human MFH share common genomic features.

**Table 1:** GSEA results for the *LSL-Kras^G12D^; Trp53^Flox/Flox^* geneset derived from the mouse model of soft tissue sarcoma (Table S3).

	Nakayama [10]	Detwiller [9]	Baird [8]
**Malignant Fibrous Histiocytoma**	**0.001 (0.012)**	**0.014 (0.149)**	**0.024 (0.190)**
Myxofibrosarcoma	0.162 (0.559)	-	-
Fibrosarcoma	0.177 (0.326)	0.159 (0.784)	DNE
Leiomyosarcoma	0.262 (0.673)	0.854 (0.981)	0.401 (0.715)
Synovial Sarcoma	DNE	DNE	DNE
Myxoid Liposarcoma	DNE	-	-
Dedifferentiated Liposarcoma	DNE	-	-
Rhabdomyosarcoma	-	-	0.586 (0.781)
Ewing's Sarcoma	-	-	DNE

The mouse sarcoma geneset was used to examine three human soft tissue sarcoma datasets [8], [9], [10]. Only undifferentiated pleomorphic sarcoma/MFH demonstrated statistically significant enrichment. Table denotes p-values with FDR in parentheses. Bolded results note significance with p<0.05; FDR<0.25. DNE = Did Not Enrich, dash marks represent insufficient data points to do comparison.

### Ras Pathway Activity Is Enriched in Human MFH

**Figure 2:**
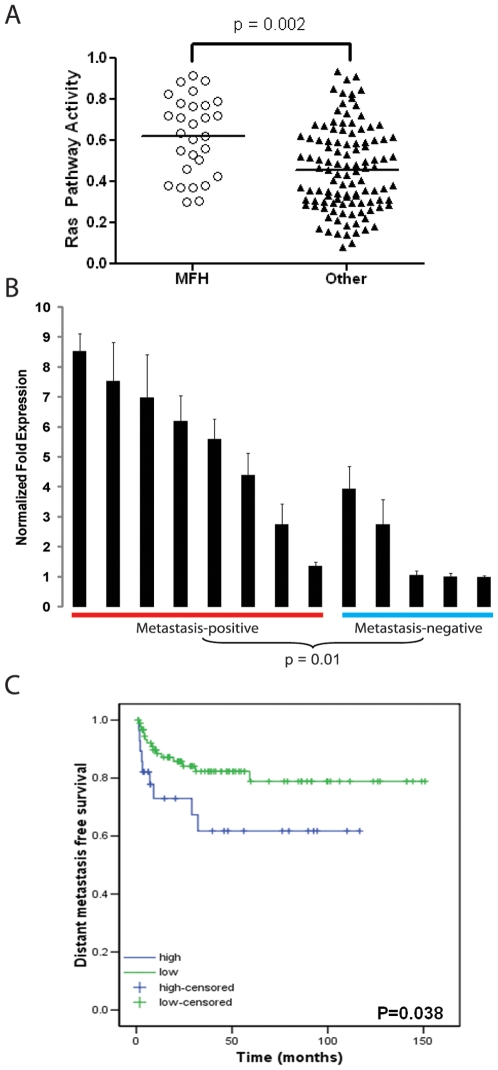
The *LSL-Kras^G12D^*; *Trp53^Flox/Flox^* mouse model of soft tissue sarcoma provides insight into human MFH. *A*, An oncogenic Ras signature is enriched in human MFH samples compared to other types of soft tissue sarcoma (p = 0.002, non-parametric Mann-Whitney test). *B*, Q-RT-PCR for *Foxm1* in murine soft tissue sarcomas correlates with metastatic potential of primary tumors (p = 0.01, two-tailed student's T-test, scale bars represent one standard deviation). *C*, FOXM1 expression in a tissue microarray correlates with metastasis free survival in human MFH (p = 0.038).

The initiating events of human MFH are not well understood. Previous studies have shown mutations in p53 occur in 36% of human MFH [15] while the rate of canonical *RAS* mutations in human MFH varies from 0–50% [16], [17]. We hypothesized that the RAS pathway may be activated in human MFH even in the absence of canonical RAS mutations. To explore a link between MFH and Ras, we utilized previously described oncogenic pathway predictors that correlate with *RAS* activity [13]. The Ras oncogenic signature is enriched in human MFH samples compared to a panel of other soft tissue sarcomas (p = 0.002) (Fig. 2A). Moreover, in human MFH (n = 8) lacking canonical RAS mutations, we observed nuclear staining of phospho-ERK by immunohistochemistry in greater than 30% of tumor cells in 7 of 8 samples (Fig. S2).

### FoxM1 Is a Novel Marker of Metastasis in MFH

Because human MFH and the *LSL-Kras^G12D^; Trp53^Flox/Flox^* mouse model of soft tissue sarcoma share common genomic features, we wanted to determine if these shared features could be used to identify diagnostic or prognostic factors for human MFH. As MFH is considered a diagnosis of exclusion, we initially attempted to identify a marker that is specific to MFH. We identified a panel of 10 candidate biomarkers based on their common upregulation in both human MFH and the mouse sarcoma model (Table S5). Expression of 9 of these candidates was validated in an independent cohort of mouse sarcomas by Q-RT-PCR (Fig. S3).

FOXM1, which is a member of the forkhead transcription factor family that enhances tumorigenesis in other solid tumors [18], was selected for further analysis because it is downstream of *Ras* [19]. Immunohistochemistry for FOXM1 in a panel of 8 human MFH samples demonstrated nuclear staining for FOXM1 in all 8 tumors (Fig. S2). We next analyzed the expression of FOXM1 in a clinically annotated tissue microarray (TMA) containing 166 MFH samples and 48 other soft tissue sarcomas. Although 84% of the MFH samples stained positive for FOXM1 (p = 0.02, Fig. S4), this marker was also expressed, but to a lesser degree, in other sarcoma subtypes. Therefore, FOXM1 may not be a useful diagnostic marker for human MFH.

Because FoxM1 has previously been shown to regulate the expression of matrix metalloproteases MMP-2 and -9, which are key mediators of cell invasion [20], we hypothesized that high FOXM1 expression may correlate with metastasis. We measured *Foxm1* gene expression in murine soft tissue sarcomas and found a correlation with the development of lung metastases (Fig. 2B, p = 0.01). Likewise, human MFH with high FOXM1 expression correlated with decreased metastasis-free survival compared to sarcomas with low to no FOXM1 expression (Fig. 2C). In contrast, overexpression of another candidate marker MELK (Table S5) did not correlate with metastasis-free survival (Fig. S5).

## Discussion

We used cross species genomic analysis to determine which human sarcoma subtype is represented by the *LSL-Kras^G12D^; Trp53^Flox/Flox^* mouse model of soft tissue sarcoma. We identified a geneset in the mouse sarcomas that is highly enriched in human MFH. This geneset was not enriched in other human sarcomas, such as fibrosarcoma or leiomyosarcoma, which can be difficult to distinguish from MFH. Furthermore, we have identified enrichment of Ras pathway activity in human MFH compared to other types of soft-tissue sarcoma. Additionally, the *LSL-Kras^G12D^; Trp53^Flox/Flox^* mouse model of soft tissue sarcoma has a propensity to metastasize to the lungs much like human MFH [5]. Based on this genomic analysis, the pattern of lung metastasis, and the similarity of the mouse sarcomas to human MFH at the histological level (Fig. S6), we conclude that this model closely resembles MFH.

We recognize that the diagnosis of undifferentiated pleomorphic sarcoma (MFH) has recently been questioned as a distinct clinical entity [2], [3], [4]. Our results do not exclude the possibility that MFH is a collection of mesenchymal tumors derived from different cell types that share an undifferentiated state. However, our finding of a sarcoma geneset conserved between mouse sarcomas and human MFH suggests that this subtype of human sarcoma shares an underlying biology beyond a common histologic appearance. Moreover, the use of cross-species analysis to identify FOXM1 as a marker of metastasis-free survival in human MFH supports the use of this mouse model to understand mechanisms of metastasis, to investigate the cell(s) of origin, and to develop novel therapies for human MFH.

## Supporting Information

Supplementary Methods S1 Supplementary Methods
(0.07 MB DOC)

Table S1 Geneset derived from mouse model of synovial sarcoma versus control (normal muscle). Geneset was derived using signal-to-noise metric.
(0.04 MB DOC)

Table S2 GSEA results for synovial sarcoma geneset derived from mouse model of synovial sarcoma (Table S1). The mouse synovial sarcoma geneset was used to examine three datasets of human soft tissue sarcomas. Table denotes p-values with FDR in parentheses. Bolded results note significance with p<0.05; FDR<0.25. DNE = Did Not Enrich, dash marks represent insufficient data points to do comparison.
(0.04 MB DOC)

Table S3 Geneset derived from *LSL-Kras^G12D^; Trp53^Flox/Flox^* mouse model of soft tissue sarcoma compared to control (normal muscle). Genes were identified using signal-to-noise metric with the top 100 genes used in the geneset.
(0.04 MB DOC)

Table S4 Geneset used in Figure 1c was derived from Nakayama et al [10] using signal-to-noise metric comparing MFH versus control (other soft tissue sarcomas).
(0.05 MB DOC)

Table S5 Candidate marker genes of human MFH were identified using differentially expressed genes between human MFH and other sarcomas (T-test, p<0.001) [9]–[10]. This list of genes was then cross referenced against the *LSL-Kras^G12D^; Trp53^Flox/Flox^* soft tissue sarcoma geneset (Table S3) and ten overlapping genes were identified.
(0.03 MB DOC)

Figure S1 The mouse synovial sarcoma geneset (Table S1) shows strong enrichment for human synovial sarcoma (Enrichment Score (ES) = 0.67, Normalized Enrichment Score (NES) = 2.21) in a human soft tissue sarcoma dataset[10] (p = 0.00024; FDR = 0.004).
(0.21 MB PDF)

Figure S2 Representative immunohistochemistry for A phospho-ERK and B FOXM1 show strong nuclear staining from the same human tumor sample. All scale bars represent 100 microns.
(9.18 MB PDF)

Figure S3 Box and whisker plots of nine candidate genes selected for Q-RT-PCR using an independent set of mouse soft tissue sarcomas (n = 5) and normal muscle samples (n = 3). Relative fold expression determined to lowest expressing normal muscle sample. Significance of differentially expressed genes was determined by two-tailed student's T-test.
(0.31 MB PDF)

Figure S4 Immunostaining of tissue microarrays (TMAs) for FOXM1 and MELK identifies expression in MFH. Tissue Microarrays (TMAs) containing 214 soft tissue sarcomas were stained for FOXM1 and scored semi-quantitatively. The degree of staining for A FOXM1 (p = 0.0017, non-parametric Mann-Whitney test) and B MELK (p = 0.02, non-parametric Mann-Whitney test) was correlated with MFH.
(0.20 MB PDF)

Figure S5 Potential biomarker MELK does not correlate with metastasis free survival (p = 0.46) in MFH patients. MFH patients were segregated based on high (3+) or low (0-2+) staining on TMAs and survival of the cohorts was compared by Kaplan-Meier analysis.
(0.28 MB PDF)

Figure S6 The *LSL-Kras^G12D^; Trp53^Flox/Flox^* mouse model of soft tissue sarcoma mimics human undifferentiated pleomorphic sarcoma/Malignant Fibrous Histiocytoma (MFH). A, The gross appearance of mouse sarcomas includes areas of necrosis and hemorrhage. The microscopic appearance of these tumors includes high grade sarcomas with B spindle cells and C more pleomorphic cells with D epitheloid like cells and frequent atypical nuclei (arrow). All scale bars represent 100 µm.
(1.05 MB JPG)

## References

[1] Ozzello L, Stout AP, Murray MR (1963). Cultural characteristics of malignant histiocytomas and fibrous xanthomas.. Cancer.

[2] Fletcher CD (1992). Pleomorphic malignant fibrous histiocytoma: fact or fiction? A critical reappraisal based on 159 tumors diagnosed as pleomorphic sarcoma.. Am J Surg Pathol.

[3] Fletcher CD, Gustafson P, Rydholm A, Willen H, Akerman M (2001). Clinicopathologic re-evaluation of 100 malignant fibrous histiocytomas: prognostic relevance of subclassification.. J Clin Oncol.

[4] Hollowood K, Fletcher CD (1995). Malignant fibrous histiocytoma: morphologic pattern or pathologic entity?. Semin Diagn Pathol.

[5] Kirsch DG, Dinulescu DM, Miller JB, Grimm J, Santiago PM (2007). A spatially and temporally restricted mouse model of soft tissue sarcoma.. Nat Med.

[6] Bolstad BM, Irizarry RA, Astrand M, Speed TP (2003). A comparison of normalization methods for high density oligonucleotide array data based on variance and bias.. Bioinformatics.

[7] Irizarry RA, Hobbs B, Collin F, Beazer-Barclay YD, Antonellis KJ (2003). Exploration, normalization, and summaries of high density oligonucleotide array probe level data.. Biostatistics.

[8] Baird K, Davis S, Antonescu CR, Harper UL, Walker RL (2005). Gene expression profiling of human sarcomas: insights into sarcoma biology.. Cancer Res.

[9] Detwiller KY, Fernando NT, Segal NH, Ryeom SW, D'Amore PA (2005). Analysis of hypoxia-related gene expression in sarcomas and effect of hypoxia on RNA interference of vascular endothelial cell growth factor A.. Cancer Res.

[10] Nakayama R, Nemoto T, Takahashi H, Ohta T, Kawai A (2007). Gene expression analysis of soft tissue sarcomas: characterization and reclassification of malignant fibrous histiocytoma.. Mod Pathol.

[11] Subramanian A, Tamayo P, Mootha VK, Mukherjee S, Ebert BL (2005). Gene set enrichment analysis: a knowledge-based approach for interpreting genome-wide expression profiles.. Proc Natl Acad Sci U S A.

[12] Johnson WE, Li C, Rabinovic A (2007). Adjusting batch effects in microarray expression data using empirical Bayes methods.. Biostatistics.

[13] Bild AH, Yao G, Chang JT, Wang Q, Potti A (2006). Oncogenic pathway signatures in human cancers as a guide to targeted therapies.. Nature.

[14] Haldar M, Hancock JD, Coffin CM, Lessnick SL, Capecchi MR (2007). A conditional mouse model of synovial sarcoma: insights into a myogenic origin.. Cancer Cell.

[15] Leach FS, Tokino T, Meltzer P, Burrell M, Oliner JD (1993). p53 Mutation and MDM2 amplification in human soft tissue sarcomas.. Cancer Res.

[16] Yoo J, Robinson RA (1999). H-ras and K-ras mutations in soft tissue sarcoma: comparative studies of sarcomas from Korean and American patients.. Cancer.

[17] Yoo J, Robinson RA, Lee JY (1999). H-ras and K-ras gene mutations in primary human soft tissue sarcoma: concomitant mutations of the ras genes.. Mod Pathol.

[18] Liu M, Dai B, Kang SH, Ban K, Huang FJ (2006). FoxM1B is overexpressed in human glioblastomas and critically regulates the tumorigenicity of glioma cells.. Cancer Res.

[19] Ma RY, Tong TH, Cheung AM, Tsang AC, Leung WY (2005). Raf/MEK/MAPK signaling stimulates the nuclear translocation and transactivating activity of FOXM1c.. J Cell Sci.

[20] Wang IC, Chen YJ, Hughes DE, Ackerson T, Major ML (2008). FoxM1 regulates transcription of JNK1 to promote the G1/S transition and tumor cell invasiveness.. J Biol Chem.

