# Supplementary material for: Cross Species Genomic Analysis Identifies a Mouse Model as Undifferentiated Pleomorphic Sarcoma/Malignant Fibrous Histiocytoma
Source: PLoS One. 2009 Nov 30;4(11):e8075. doi: 10.1371/journal.pone.0008075 (PMC2779485; doi:10.1371/journal.pone.0008075)
Supplement: Supplementary Methods S1 — Supplementary Methods (0.07 MB DOC) [file pone.0008075.s001.doc]

**Supplementary methods**

**Mouse Genotyping, Tumor Generation, and Determination of Metastatic Potential**

Both mouse genotyping and generation of tumors was carried out as described previously[1] in accordance with Duke University and MIT Institutional Animal Care and Use Committee approved protocols. Sarcomas were induced in the lower left limb and allowed to grow until ~200mm^3^ in volume. Tumors were then surgically excised via amputation of the limb and animals followed for a minimum of 4 months to determine the metastatic potential of the primary tumor. Tumors or normal muscle were isolated and stored in RNA later (Ambion) prior to RNA isolation.

**RNA isolation**

RNA was extracted from *LSL-Kras^G12D^; Trp53^Flox/Flox^* tumors or normal muscle using TRIzol reagent (Invitrogen) and was purified using RNeasy mini kit (Qiagen) per manufacturer’s instructions.

**Microarray Processing and Analysis**

Gene expression was determined using Affymetrix 430A 2.0 arrays (Affymetrix). Labeled RNA was prepared from 2 µg total RNA using one-cycle target labeling kit (Affymetrix). cRNA was fragmented, hybridized to arrays, and scanned on the GeneChip Scanner 3000 (Affymetrix). Data from GeneChip Scanner was analyzed using GeneChip Command Console software v1.1 (Affymetrix) to generate .CEL files.

.CEL files were processed using the RMA algorithm[2,3,4] to normalize the data. Then, datasets were converted into .gct files for use in GSEA.

Human and Mouse datasets[5,6,7] were downloaded from GEO (GSE6461, GSE6481, GSE2553, and GDS1209), normalized with RMA (when appropriate), and converted into .gct files for use in GSEA.

Genesets were identified using a signal-to-noise metric (μ_class 0_ - μ_class 1_)/(σ_class 0_ + σ_class 1_) where µ and σ represent the mean and standard deviation respectively for each class.

GSEA was performed by permutation of the phenotype labels 10,000 times, with weighted enrichment statistic. Genes were ranked using the signal-to-noise metric, multiple probes per gene were collapsed to the highest expressing probe. All analyses were performed in this manner unless noted otherwise. Data for human to mouse comparison was permuted with 1000 random permutations of the genesets by placing the human MFH geneset within the curated genesets database (C2 v2.5) from MsigDB[8]. Classes were defined as one soft tissue sarcoma type versus controls (other sarcoma or normal muscle) present in their respective datasets.

**Oncogenic pathway predictors**

Human soft tissue sarcoma datasets[6,7] were combined using ComBat[9] and normal tissue samples removed from the combined dataset. An oncogenic pathway classifier for Ras pathway activity was developed as described previously[10]. This classifier was used to compare undifferentiated pleiomorphic sarcoma/MFH samples (n=29) against all other soft tissue sarcomas (n=111 from 11 other soft tissue sarcoma sub-types including 20 synovial sarcomas, 19 myxoliposarcomas, 19 dedifferentiated liposarcomas, 15 myxofibrosarcomas, 12 leiomyosarcomas, 11 fibrosarcomas, 4 round cell tumors, 3 pleomorphic liposarcomas, 3 well differentiated liposarcomas, 3 malignant peripheral nerve sheath tumors, and two gastrointestinal stromal tumors). Significance was determined using a non-parametric Mann-Whitney test.

**Quantitative RT-PCR (Q-RT-PCR)**

RNA isolated from an independent cohort of tumors and normal muscle samples was isolated using TRIzol (Invitrogen). Reverse transcription reaction was performed using 500µg of total RNA and the iScript cDNA synthesis kit (Biorad) per manufacturer’s instructions. Q-RT-PCR was performed using the following primer pairs: Bcat1 5’-CTTTGGAAGGCTTCTTGACG-3’ and 5’-ACAGATCGACCAAGAATGGG-3', Ccnb1 5’-GGCTTGGAGAGGGATTATCA-3’ and 5’-ACCAGAGGTGGAACTTGCTG-3’, Ccnb2 5’- CAGAGAAAGCTTGGCAGAGG-3’ and 5’- TGAAACCAGTGCAGATGGAG-3’, Cenpa 5’- TCTGCAGGGTCTTGATTTCC-3’ and 5’- AGACCCCAAGGAGGAGACC-3’, Cenpe 5’- CCTGAAGCACTTTTCTCGAAG-3’ and 5’- GGACAACCTGAGAGAAGCCA-3’, Lpxn 5’- TTTGGCTCTTGGACCTCACT-3’ and 5’- AAAGACCTTGTCATCGCAGG, Foxm1 5’- CAGACACAGAGTCCTGCCAA-3’ and 5’- AGATGAGTTCTGACGGGCTG-3’, Marcksl1 5’- GCTTCTCACGTGGCCATT-3’ and 5’- GGCAGCCAGAGCTCTAAGG-3’, and Melk 5’- CCAGGCGAGTTGTAGTCACA-3’ and 5’- GTTCTCACTGCGCTCACAAG-3’. Primers used for Q-RT-PCR of candidate marker genes were selected from the qPrimerDepot database [11]. PCR was performed with POWER SYBR green PCR Master Mix (Applied Biosystems) and the following cycling parameters: 10 mins at 95^o^C, Forty cycles of 30 secs at 95^o^C, 15 secs at 58^o^C, and 30 secs at 72^o^C on the IQ5 Multicolor Real-Time PCR Detection System (Biorad). Samples were normalized to 18s ribosomal RNA. Relative fold expression was determined to the lowest expressing sample and differential expression was tested using a two tailed student’s T-test.

**Histology and Immunohistochemistry and image analysis**

All human samples were obtained and used in accordance with Duke and MD Anderson Cancer Center Institutional Review Board (IRB) approved protocols. Five micron thick sections were cut from formalin fixed paraffin embedded samples (FFPE). Samples were subjected to standard hematoyxlin and eosin staining or immunohistochemistry. Immunohistochemistry was performed with the following antibodies: phospho-ERK (Invitrogen 19-2389) and FOXM1 (Abcam ab47808) using the Vectastain ABC Rabbit IgG kit with Vectastain Elite ABC Reagent (Vector Labs).

Brightfield images of slides taken at 40x were used for analysis using Image Pro AMS v6.1. The counting module was trained using both positive and negative nuclear staining for phospho-ERK. A minimum of 3000 nuclei were counted per sample and a ratio between total nuclei with positive nuclei to total nuclei was determined using a minimal and maximal area of 100 and 1000 pixels respectively.

**Tissue Microarrays (TMAs)**

TMAs were generated at MD Anderson Cancer Center and contained a clinically annotated set of 214 soft tissue sarcoma samples including: 166 MFH/Unclassified sarcomas, 19 synovial sarcomas, 6 leiomyosarcomas, 8 pleomorphic liposarcomas, 8 myxoid liposarcomas, 6 atypical lipomatous tumors, and 1 dedifferentiated liposarcoma. TMAs were stained as above and scored semiquantitatively on a scale from 0-3+ by a musculoskeletal pathologist blinded to patient outcome. Scores were correlated with both diagnosis and clinical outcome.

**Statistical Analysis of TMAs**

Scoring of TMAs was correlated with diagnosis, and metastasis-free survival.

Correlation of diagnosis data was tested for normality using a chi-square test. Having not reached statistical significance, comparison between MFH and other soft tissue sarcomas was performed using the non-parametric Mann-Whitney test.

Metastasis free survival analysis was performed on MFH samples comparing 3+ staining to 0-2+ staining. Survival was determined by Kaplan-Meier analysis.

**References**

1. Kirsch DG, Dinulescu DM, Miller JB, Grimm J, Santiago PM, et al. (2007) A spatially and temporally restricted mouse model of soft tissue sarcoma. Nat Med 13: 992-997.

2. Bolstad BM, Irizarry RA, Astrand M, Speed TP (2003) A comparison of normalization methods for high density oligonucleotide array data based on variance and bias. Bioinformatics 19: 185-193.

3. Irizarry RA, Bolstad BM, Collin F, Cope LM, Hobbs B, et al. (2003) Summaries of Affymetrix GeneChip probe level data. Nucleic Acids Res 31: e15.

4. Irizarry RA, Hobbs B, Collin F, Beazer-Barclay YD, Antonellis KJ, et al. (2003) Exploration, normalization, and summaries of high density oligonucleotide array probe level data. Biostatistics 4: 249-264.

5. Baird K, Davis S, Antonescu CR, Harper UL, Walker RL, et al. (2005) Gene expression profiling of human sarcomas: insights into sarcoma biology. Cancer Res 65: 9226-9235.

6. Detwiller KY, Fernando NT, Segal NH, Ryeom SW, D'Amore PA, et al. (2005) Analysis of hypoxia-related gene expression in sarcomas and effect of hypoxia on RNA interference of vascular endothelial cell growth factor A. Cancer Res 65: 5881-5889.

7. Nakayama R, Nemoto T, Takahashi H, Ohta T, Kawai A, et al. (2007) Gene expression analysis of soft tissue sarcomas: characterization and reclassification of malignant fibrous histiocytoma. Mod Pathol 20: 749-759.

8. Subramanian A, Tamayo P, Mootha VK, Mukherjee S, Ebert BL, et al. (2005) Gene set enrichment analysis: a knowledge-based approach for interpreting genome-wide expression profiles. Proc Natl Acad Sci U S A 102: 15545-15550.

9. Johnson WE, Li C, Rabinovic A (2007) Adjusting batch effects in microarray expression data using empirical Bayes methods. Biostatistics 8: 118-127.

10. Bild AH, Yao G, Chang JT, Wang Q, Potti A, et al. (2006) Oncogenic pathway signatures in human cancers as a guide to targeted therapies. Nature 439: 353-357.

11. Cui W, Taub DD, Gardner K (2007) qPrimerDepot: a primer database for quantitative real time PCR. Nucleic Acids Res 35: D805-809.
