# Supplementary material for: Cross Species Genomic Analysis Identifies a Mouse Model as Undifferentiated Pleomorphic Sarcoma/Malignant Fibrous Histiocytoma
Source: PLoS One. 2009 Nov 30;4(11):e8075. doi: 10.1371/journal.pone.0008075 (PMC2779485; doi:10.1371/journal.pone.0008075)
Supplement: Table S1 — Geneset derived from mouse model of synovial sarcoma versus control (normal muscle). Geneset was derived using signal-to-noise metric. (0.04 MB DOC) [file pone.0008075.s002.doc]

| **Mouse Synovial Sarcoma Geneset** | | |  |  |
| --- | --- | --- | --- | --- |
|  |  |  |  |  |
| *2610039C10Rik* | *Cpt1a* | *Gsta4* | *Ngfrap1* | *Sh3bgrl2* |
| *5830411G16Rik* | *Ctbp2* | *Hmgn3* | *Nipsnap1* | *Slc12a7* |
| *9630031F12Rik* | *Cyp51a1* | *Igsf4* | *Nlgn2* | *Slc39a6* |
| *Aldh18a1* | *Dna2l* | *Il17rc* | *Nme7* | *Smo* |
| *Aplp1* | *Dtx3* | *Inadl* | *Nr2f1* | *Tjp2* |
| *Aw146242* | *Efna3* | *Kcnk1* | *Nt5dc2* | *Tle3* |
| *Bcl11a* | *Ephb2* | *Kif23* | *Pbk* | *Tmem132a* |
| *Bmp7* | *Ephb3* | *Lhx1* | *Pcdhb3* | *Tmem30b* |
| *Cachd1* | *Evl* | *Lhx8* | *Pde9a* | *Tmsb10* |
| *Cacna1g* | *Fkbp10* | *Limd2* | *Prim1* | *Tnc* |
| *Cbx4* | *Fkbp1b* | *LOC630539* | *Ptprk* | *Trim37* |
| *Ccna2* | *Fndc4* | *LOC673890* | *Ptprs* | *Trim46* |
| *Ccnd1* | *Gad1* | *Man2c1* | *Purg* | *Trip6* |
| *Cdc7* | *Gcs1* | *Mdk* | *Rad18* | *Ttc3* |
| *Cdk4* | *Gli2* | *Melk* | *Rasl11b* | *Tuba3* |
| *Cdk4* | *Gmcl1* | *Mfap2* | *Rbbp8* | *Tubb* |
| *Chst2* | *Gmpr2* | *Msh6* | *Rfc5* | *Wbp5* |
| *Clcn5* | *Gpm6b* | *Msx1* | *Rgs19* | *Wnt5a* |
| *Col16a1* | *Gprasp1* | *Nfatc4* | *Sall2* | *Zfp90* |
| *Col18a1* | *Grik5* | *Ngfr* | *Scube2* | *Zic5* |
