# Supplementary material for: Cross Species Genomic Analysis Identifies a Mouse Model as Undifferentiated Pleomorphic Sarcoma/Malignant Fibrous Histiocytoma
Source: PLoS One. 2009 Nov 30;4(11):e8075. doi: 10.1371/journal.pone.0008075 (PMC2779485; doi:10.1371/journal.pone.0008075)
Supplement: Table S2 — GSEA results for synovial sarcoma geneset derived from mouse model of synovial sarcoma (Table S1). The mouse synovial sarcoma geneset was used to examine three datasets of human soft tissue sarcomas. Table denotes p-values with FDR in parentheses. Bolded results note significance with p<0.05; FDR<0.25. DNE = Did Not Enrich, dash marks represent insufficient data points to do comparison. (0.04 MB DOC) [file pone.0008075.s003.doc]

| **Tumor Type** | **Nakayama[1]** | **Detwiller[2]** | **Baird[3]** |
| --- | --- | --- | --- |
| Malignant Fibrous Histiocytoma | DNE | DNE | DNE |
| Myxofibrosarcoma | DNE | - | - |
| Fibrosarcoma | DNE | - | - |
| Leiomyosarcoma | DNE | DNE | 0.655 (0.806) |
| **Synovial Sarcoma** | **0.00024 (0.004)** | 0.003 (0.371) | 0.008 (0.471) |
| Myxoid Liposarcoma | DNE | - | - |
| Dedifferentiated Liposarcoma | DNE | - | - |
| Rhabdomyosarcoma | - | - | 0.453 (0.684) |
| Ewing's Sarcoma | - | - | 0.630 (0.921) |
