# Supplementary material for: Cross Species Genomic Analysis Identifies a Mouse Model as Undifferentiated Pleomorphic Sarcoma/Malignant Fibrous Histiocytoma
Source: PLoS One. 2009 Nov 30;4(11):e8075. doi: 10.1371/journal.pone.0008075 (PMC2779485; doi:10.1371/journal.pone.0008075)
Supplement: Table S3 — Geneset derived from LSL-KrasG12D; Trp53Flox/Flox mouse model of soft tissue sarcoma compared to control (normal muscle). Genes were identified using signal-to-noise metric with the top 100 genes used in the geneset. (0.04 MB DOC) [file pone.0008075.s004.doc]

| ***LSL-Kras^G12D^; Trp53^Flox/Flox^* Mouse Model of Soft Tissue Sarcoma Geneset** | | | | |
| --- | --- | --- | --- | --- |
|  |  |  |  |  |
| *2700099C18Rik* | *Ccr1* | *Dbf4* | *Lhx8* | *Plscr1* |
| *2810417H13Rik* | *Ccr2* | *Depdc1a* | *LOC630539* | *Plscr2* |
| *2810433K01Rik* | *Ccr5* | *Dtl* | *Lpxn* | *Pmaip1* |
| *9030408N13Rik* | *Cd109* | *Ect2* | *Marcksl1* | *Prc1* |
| *Agtr2* | *Cdc2a* | *Edil3* | *Mcm5* | *Psat1* |
| *Ai504432* | *Cdc6* | *Ereg* | *Mcm6* | *Rbp1* |
| *Aif1* | *Cdca1* | *Esm1* | *Melk* | *Rin1* |
| *Anln* | *Cdca5* | *Fcgr1* | *Mki67* | *Rrm2* |
| *Bcat1* | *Cdh11* | *Fignl1* | *Mmp13* | *Shcbp1* |
| *Birc5* | *Cdkn2a* | *Foxm1* | *Mmp3* | *Slc25a24* |
| *Bub1* | *Cenpa* | *Gp49a* | *Ms4a11* | *Smc2* |
| *C130076O07Rik* | *Cenpe* | *Gpr34* | *Ms4a6d* | *Tfpi2* |
| *C1qtnf3* | *Cep55* | *Gpr65* | *Ms4a7* | *Timp1* |
| *C79407* | *Cks2* | *Gsg2* | *Nef3* | *Top2a* |
| *Ccdc99* | *Clec4n* | *Hells* | *Nefl* | *Tpbg* |
| *Ccl12* | *Crabp1* | *Hmga2* | *Nusap1* | *Trip13* |
| *Ccl2* | *Crabp2* | *Hmgb2* | *Pbk* | *Ttk* |
| *Ccna2* | *Cx3cr1* | *Ifi44* | *Phgdh* | *Tyms* |
| *Ccnb1* | *Cxcl10* | *Iqgap3* | *Plk4* | *Zfp7* |
| *Ccnb2* | *Cybb* | *Kif20a* | *Plscr1* | *Zwilch* |
|  |  |  |  |  |
|  |  |  |  |  |
