# Supplementary material for: Cross Species Genomic Analysis Identifies a Mouse Model as Undifferentiated Pleomorphic Sarcoma/Malignant Fibrous Histiocytoma
Source: PLoS One. 2009 Nov 30;4(11):e8075. doi: 10.1371/journal.pone.0008075 (PMC2779485; doi:10.1371/journal.pone.0008075)
Supplement: Table S4 — Geneset used in Figure 1c was derived from Nakayama et al [10] using signal-to-noise metric comparing MFH versus control (other soft tissue sarcomas). (0.05 MB DOC) [file pone.0008075.s005.doc]

| **Human MFH Geneset** | |  |  |  |  |  |
| --- | --- | --- | --- | --- | --- | --- |
|  |  |  |  |  |  |  |
| *ABL2* | *CCNB1* | *EEF1E1* | *JOSD1* | *MSN* | *PTTG1* | *TFG* |
| *ACTR3* | *CCNB2* | *EFHD2* | *JTV1* | *MYBL2* | *RAB27A* | *TFRC* |
| *ADAM12* | *CCNH* | *EIF2S2* | *K-ALPHA-1* | *MYH9* | *RAN* | *THOC7* |
| *ADRM1* | *CD58* | *ENO1* | *KCMF1* | *NANS* | *RANBP1* | *TMCO1* |
| *AHCYL1* | *CDC2* | *ENPP1* | *KDELR2* | *NCAPG* | *RER1* | *TMEM50A* |
| *AKR1B1* | *CDC20* | *EVI2A* | *KDELR3* | *NOLA3* | *RIPK2* | *TNFRSF12A* |
| *ALG3* | *CDC45L* | *FEN1* | *KIAA0101* | *NRBF2* | *RPN1* | *TPMT* |
| *ANXA2* | *CDCA8* | *FLOT1* | *KIF2C* | *NUP37* | *RPP38* | *TPX2* |
| *ANXA2P2* | *CDR2* | *FOSL1* | *KIF4A* | *P4HA2* | *RPRC1* | *TRAM2* |
| *ANXA5* | *CEBPB* | *FOXM1* | *KPNA1* | *PDE4DIP* | *RRAS2* | *TTK* |
| *ANXA7* | *CENPA* | *GLRX* | *LDHA* | *PFN1* | *RRM1* | *TXNL2* |
| *AP2M1* | *CENPE* | *GLRX2* | *LGALS1* | *PGK1* | *RRM2* | *UBE2C* |
| *ARFGAP3* | *CEP55* | *GMNN* | *LOC146909* | *PHC2* | *S100A11* | *UBE2L3* |
| *ARPC2* | *CFL1* | *GNG5* | *LPXN* | *PIGT* | *S100A6* | *UCK2* |
| *ARPC5* | *CGGBP1* | *GOLT1B* | *LRRC59* | *PKM2* | *SDC4* | *UFD1L* |
| *ASPM* | *CKS2* | *GPSM2* | *M6PR* | *PLAU* | *SDF2L1* | *UGCG* |
| *ATP6V0B* | *CLIC1* | *GSPT1* | *M6PRBP1* | *PLOD2* | *SERP1* | *USP39* |
| *ATP6V0C* | *CMPK* | *GSTO1* | *MAD2L1* | *POLR2K* | *SERPINB8* | *VDAC1* |
| *AURKA* | *COMT* | *H2AFZ* | *MAGOH* | *POMP* | *SH3BGRL3* | *YARS* |
| *BAG3* | *COPG* | *HDGF* | *MAPK1* | *PPP2CA* | *SH3GLB1* | *YBX1* |
| *BIRC5* | *CORO1C* | *HLA-DPA1* | *MAPRE1* | *PRDX1* | *SIAH2* |  |
| *BUB3* | *CREB3L1* | *HMMR* | *MCM4* | *PSMA1* | *SMC4* |  |
| *C1ORF144* | *CRKL* | *HNRPAB* | *MELK* | *PSMA2* | *SMS* |  |
| *C20ORF24* | *CYCS* | *HPRT1* | *MICAL2* | *PSMA5* | *SNRPA1* |  |
| *C6ORF79* | *D15WSU75E* | *HSPA1A* | *MICAL-L1* | *PSMB2* | *SRM* |  |
| *CALU* | *DAP* | *HSPA1B* | *MICB* | *PSMC2* | *STK17A* |  |
| *CAP1* | *DEK* | *HSPC171* | *MIF* | *PSMD11* | *STK17B* |  |
| *CAPZB* | *DEPDC1* | *IBRDC3* | *MKI67* | *PSMD14* | *TAF10* |  |
| *CCDC109B* | *DR1* | *ICMT* | *MME* | *PSMD2* | *TAGLN2* |  |
| *CCNA2* | *DRAP1* | *IMPAD1* | *MRPL3* | *PTP4A2* | *TARS* |  |
