# Supplementary material for: Cross Species Genomic Analysis Identifies a Mouse Model as Undifferentiated Pleomorphic Sarcoma/Malignant Fibrous Histiocytoma
Source: PLoS One. 2009 Nov 30;4(11):e8075. doi: 10.1371/journal.pone.0008075 (PMC2779485; doi:10.1371/journal.pone.0008075)
Supplement: Table S5 — Candidate marker genes of human MFH were identified using differentially expressed genes between human MFH and other sarcomas (T-test, p<0.001) [9]–[10]. This list of genes was then cross referenced against the LSL-KrasG12D; Trp53Flox/Flox soft tissue sarcoma geneset (Table S3) and ten overlapping genes were identified. (0.03 MB DOC) [file pone.0008075.s006.doc]

| **Candidate Markers** |
| --- |
| BCAT1 |
| CCNB1 |
| CCNB2 |
| CENPA |
| CENPE |
| FCGR1 |
| FOXM1 |
| LPXN |
| MARCKSL1 |
| MELK |
