# Supplementary figures and images for: Cross Species Genomic Analysis Identifies a Mouse Model as Undifferentiated Pleomorphic Sarcoma/Malignant Fibrous Histiocytoma
Source: PLoS One. 2009 Nov 30;4(11):e8075. doi: 10.1371/journal.pone.0008075 (PMC2779485; doi:10.1371/journal.pone.0008075)

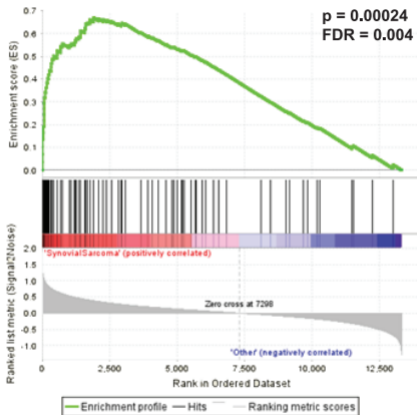

Supplement: Figure S1 — The mouse synovial sarcoma geneset (Table S1) shows strong enrichment for human synovial sarcoma (Enrichment Score (ES) = 0.67, Normalized Enrichment Score (NES) = 2.21) in a human soft tissue sarcoma dataset[10] (p = 0.00024; FDR = 0.004). (0.21 MB PDF) [file pone.0008075.s007.pdf]

A

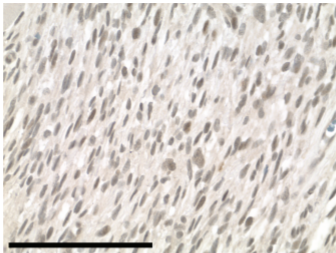

B

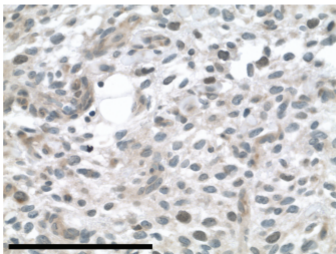

Supplement: Figure S2 — Representative immunohistochemistry for A phospho-ERK and B FOXM1 show strong nuclear staining from the same human tumor sample. All scale bars represent 100 microns. (9.18 MB PDF) [file pone.0008075.s008.pdf]

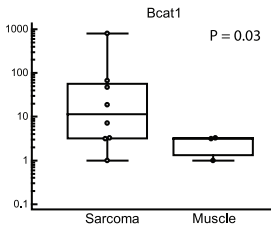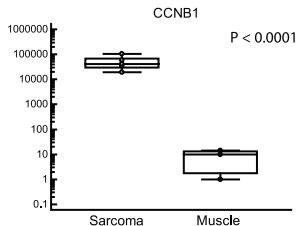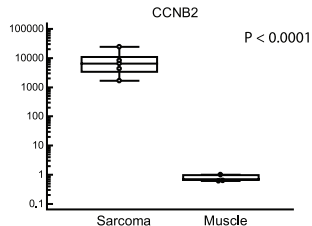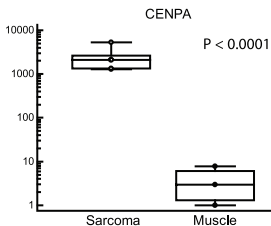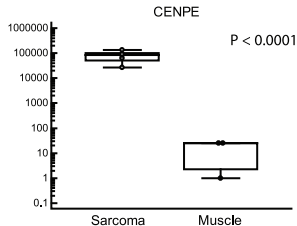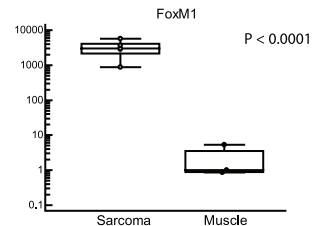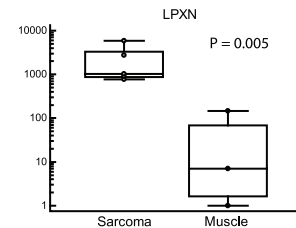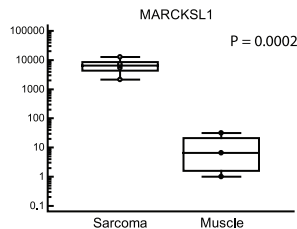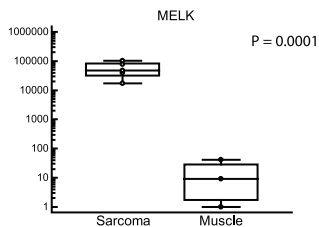

Supplement: Figure S3 — Box and whisker plots of nine candidate genes selected for Q-RT-PCR using an independent set of mouse soft tissue sarcomas (n = 5) and normal muscle samples (n = 3). Relative fold expression determined to lowest expressing normal muscle sample. Significance of differentially expressed genes was determined by two-tailed student's T-test. (0.31 MB PDF) [file pone.0008075.s009.pdf]

A

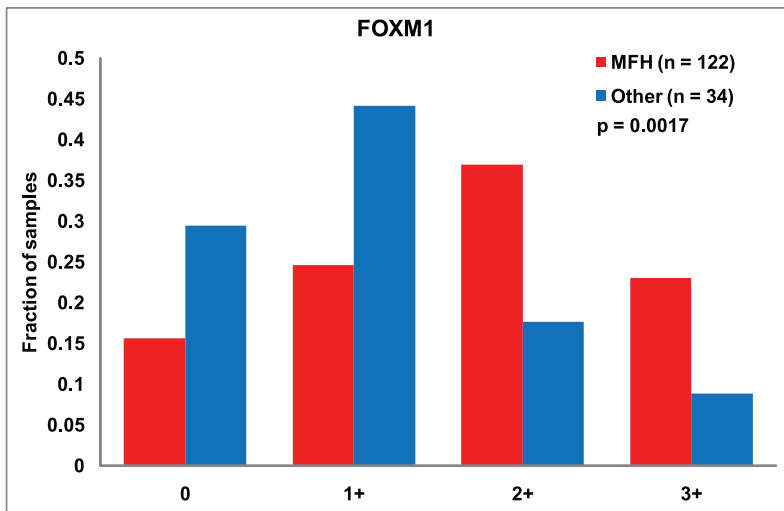

B

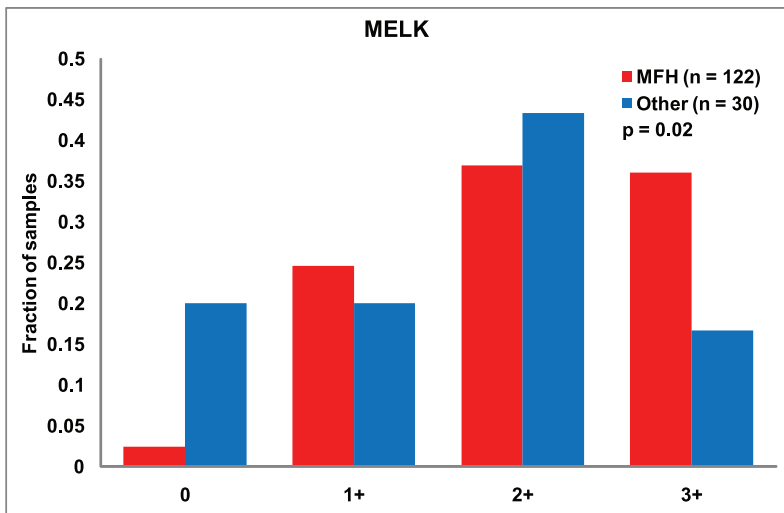

Supplement: Figure S4 — Immunostaining of tissue microarrays (TMAs) for FOXM1 and MELK identifies expression in MFH. Tissue Microarrays (TMAs) containing 214 soft tissue sarcomas were stained for FOXM1 and scored semi-quantitatively. The degree of staining for A FOXM1 (p = 0.0017, non-parametric Mann-Whitney test) and B MELK (p = 0.02, non-parametric Mann-Whitney test) was correlated with MFH. (0.20 MB PDF) [file pone.0008075.s010.pdf]

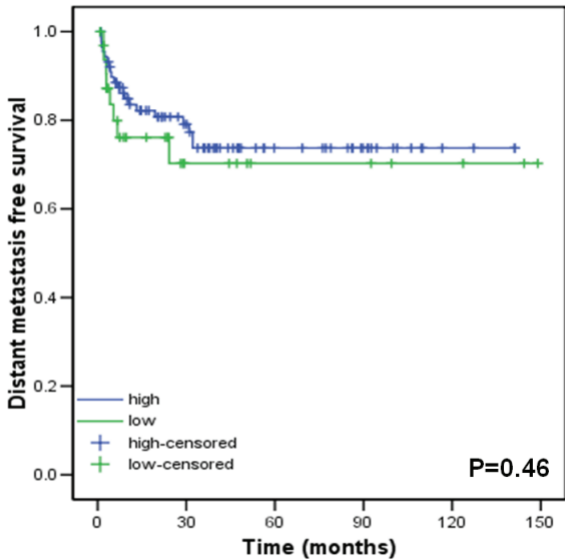

Supplement: Figure S5 — Potential biomarker MELK does not correlate with metastasis free survival (p = 0.46) in MFH patients. MFH patients were segregated based on high (3+) or low (0-2+) staining on TMAs and survival of the cohorts was compared by Kaplan-Meier analysis. (0.28 MB PDF) [file pone.0008075.s011.pdf]

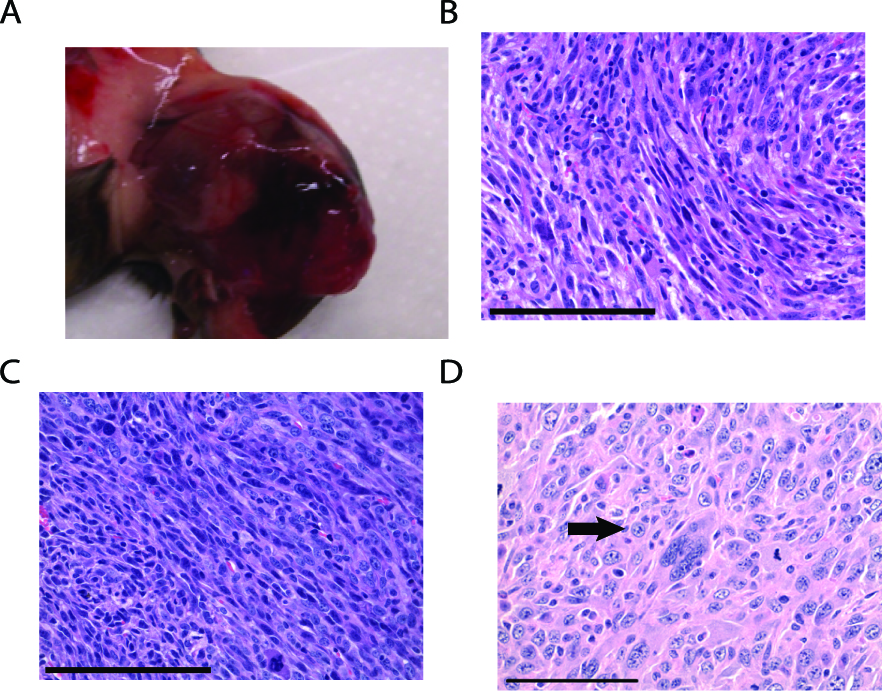

Supplement: Figure S6 — The LSL-KrasG12D; Trp53Flox/Flox mouse model of soft tissue sarcoma mimics human undifferentiated pleomorphic sarcoma/Malignant Fibrous Histiocytoma (MFH). A, The gross appearance of mouse sarcomas includes areas of necrosis and hemorrhage. The microscopic appearance of these tumors includes high grade sarcomas with B spindle cells and C more pleomorphic cells with D epitheloid like cells and frequent atypical nuclei (arrow). All scale bars represent 100 µm. (1.05 MB JPG) [file pone.0008075.s012.jpg]
